# Transdiagnostic Dimensions of Psychiatric Comorbidity in Individuals at Clinical High Risk for Psychosis: A Preliminary Study Informed by HiTOP

**DOI:** 10.3389/fpsyt.2020.614710

**Published:** 2021-01-08

**Authors:** Henry R. Cowan, Vijay A. Mittal

**Affiliations:** ^1^Department of Psychology, Northwestern University, Evanston, IL, United States; ^2^Department of Psychology, Psychiatry and Medical Social Sciences, Institute for Policy Research, Northwestern University, Evanston, IL, United States

**Keywords:** clinical high risk (CHR) for psychosis, comorbidity, factor analysis, detachment, internalizing, positive symptoms of psychosis, negative symptoms of psychosis, hierarchical taxonomy of psychopathology (HiTOP)

## Abstract

**Background:** Although psychiatric comorbidity is the norm among individuals at clinical high risk for psychotic disorders (CHR), research has yet to examine transdiagnostic dimensional models of comorbidity in this critical population.

**Methods:** This study analyzed quantitative measures of eleven psychiatric syndromes in a group at CHR (*n* = 71) and a matched healthy comparison group (*n* = 73) to determine these syndromes' dimensional structure and relationships to cognition, functioning, and risk of conversion to psychotic disorders.

**Results:** Relative to the comparison group, the CHR group was elevated on all eleven psychiatric syndromes. Exploratory factor analysis found three psychopathology dimensions: internalizing, negative symptoms, and positive symptoms. Depression cross-loaded onto the internalizing and negative symptom dimensions. Hypomania loaded positively on positive symptoms but negatively on negative symptoms. The negative symptom factor was associated with poorer cognition and functioning and a higher risk of conversion to psychosis.

**Conclusions:** These dimensions align with internalizing, detachment, and thought disorder, three of the five spectra in higher-order models such as the Hierarchical Taxonomy of Psychopathology (HiTOP). In the CHR state, detachment appears to be particularly insidious and predictive of psychosis. Further research is required to distinguish depression and hypomania from attenuated psychotic symptoms in this population.

## Introduction

Psychiatric comorbidity presents an enduring puzzle in schizophrenia. Most people diagnosed with schizophrenia also qualify for at least one other DSM diagnosis, most commonly mood, anxiety, and substance use disorders (1). Comorbidity rates are as high or higher among individuals at clinical high risk for psychotic disorders (CHR), that is, individuals without a current psychotic disorder who show elevated risk for psychosis based on attenuated psychotic symptoms, brief intermittent psychotic symptoms, or genetic risk and functional decline (2). Seventy to eighty percent of individuals in CHR studies tend to meet criteria for at least one lifetime non-psychotic DSM disorder (3–7). However, research has yet to apply transdiagnostic dimensional models of comorbidity in this critical population.

Conceptually, how can researchers and clinicians make sense of populations in which most individuals meet diagnostic criteria for multiple psychiatric disorders? The comorbidity puzzle has generated considerable debate about overlap between disorders and relationships between normative variation and psychopathology (8–10). Widely used diagnostic systems (DSM-5 and ICD-10) present two solutions: (a) diagnose multiple co-occurring, putatively independent disorders (meeting the traditional definition of comorbidity); or (b) diagnose hierarchically, such that one diagnosis can take precedence over or subsume the symptoms of another. For instance, DSM-5 and ICD-10 describe anxiety and depression as possible features of schizophrenia in addition to symptoms of co-occurring disorders (11, 12). There are advantages to both approaches, with the independent-disorders approach prioritizing full information and the hierarchical approach prioritizing parsimony.

Recently, an alternative transdiagnostic framework has emerged which models symptoms as correlated indicators of latent dimensions (8, 13–15). For instance, a latent internalizing dimension could be expressed in one case as social anxiety symptoms, in another case as social anxiety and obsessive-compulsive symptoms, and in a third case as social anxiety and panic symptoms. Multivariate dimensional models allow symptoms to be understood at multiple levels of analysis, providing both parsimony at broader levels of analysis (e.g., latent psychopathology dimensions) and full information at specific levels of analysis (e.g., manifest psychiatric syndromes). This hierarchical dimensional approach has been codified in the Hierarchical Taxonomy of Psychopathology (HiTOP), which links broad spectra (e.g., internalizing) to specific syndromes (e.g., social anxiety) through descending levels of specificity (15)^1^.

Dimensional models are widely used in schizophrenia research, most notably in the classic distinction between positive and negative symptom dimensions (16). Indeed, symptom dimensions have consistently outperformed categorical diagnoses in explaining clinical outcomes in individuals with psychotic diagnoses (16, 17). Recent research shows that a thought disorder/positive symptom dimension is clearly distinct from a detachment/negative symptom dimension; both dimensions can be further subdivided; and both dimensions relate to normative and abnormal personality processes (18–25). Most of this work has focused on the structure of psychotic symptoms, and less is known about how these symptoms fit within broader transdiagnostic models of psychopathology such as HiTOP (15).

Transdiagnostic models are increasingly relevant as psychosis research focuses on the CHR state, aiming to identify early risk indicators, understand the pathogenesis of psychotic disorders, and develop early interventions (26). Only 10–30% of CHR individuals go on to develop a psychotic disorder (26, 27), but the remaining 70–90%, traditionally classified as “nonconverters,” show persistent cognitive and functional impairment (28) and high rates of nonpsychotic disorders (5). Many researchers now adopt a clinical staging framework which models the CHR state as a transdiagnostic indicator of pooled risk for multiple disorder phenotypes (29, 30). Dimensional models can be important tools in understanding transdiagnostic elements of the CHR state: they can clarify the conceptual status of psychiatric comorbidity; use all the data at our disposal to improve prediction of psychotic disorders; and deepen our understanding of nonconverters who may instead develop chronic nonpsychotic pathology.

This preliminary study presents a transdiagnostic dimensional analysis of psychiatric comorbidity in a CHR sample. We carried out a secondary analysis of an extant dataset in which we identified continuous self-report and interview measures of eleven psychiatric syndromes covering positive symptoms, negative symptoms, internalizing, externalizing, and hypomania. We examined latent dimensions through exploratory factor analysis. Although this research was primarily exploratory, we hypothesized that the dimensions would reflect two or more of the five spectra identified in HiTOP research (detachment, thought disorder, internalizing, disinhibited externalizing, and antagonistic externalizing). We then examined the dimensions' clinical utility by analyzing their relationships to cognition, social and role functioning, and risk of conversion to a psychotic disorder. We hypothesized that psychotic dimensions would outperform nonpsychotic dimensions in predicting conversion risk, but that psychotic and nonpsychotic dimensions may both impair cognition and functioning.

## Methods and Materials

### Participants

Participants were 71 help-seeking community participants who qualified for a CHR syndrome as defined by the Structured Interview for Psychosis-Risk Syndromes (2), and 73 matched healthy comparison participants (HC). Participants were recruited at a university research clinic specializing in psychosis-risk in a midsize Western American city, through community professional referrals, newspaper, transit, and Craigslist ads, and e-mail postings. Participants in the CHR group were referred or self-referred based on unusual experiences such as suspiciousness, social withdrawal, or “mind tricks,” and distress associated with these experiences.

The CHR group was 39% (*n* = 28) female; 68% (31) White, 15% (11) Hispanic, and 17% (12) other race; with a mean age of 18.7 (SD = 1.8); a mean of 12.4 (SD = 1.8) years of education; and a median family income of $60,000–$99,999. Psychiatric prescriptions rates were 12 (17%) participants prescribed stimulant medication, 11 (15%) SSRIs, 8 (11%) antipsychotics, 8 (11%) other antidepressants, and 7 (10%) mood stabilizers. Comorbid DSM-IV-TR Axis I disorders included 21 mood disorders (29%), 6 posttraumatic stress disorder (8%), 6 obsessive compulsive disorder (8%), 25 other anxiety disorders (34%), 7 attention-deficit/hyperactivity disorder (10%), and 1 eating disorder (1%). Six participants (8.5%) converted to a confirmed psychotic disorder within 24 months: these included 2 diagnoses of psychotic disorder NOS, 1 schizophrenia, 1 schizophreniform, 1 bipolar disorder with psychotic features, and 1 brief psychotic disorder.

The HC group was 56% (32) female; 63% (33) White, 19% (14) Hispanic, and 18% (13) other race; with a mean age of 18.2 (SD = 2.6); a mean of 12.3 (SD = 2.5) years of education; and a median family income of $60,000–$99,999.

### Procedures

Participants completed clinical interviews, self-report instruments, and cognitive testing as part of a baseline assessment battery for an observational study of psychosis risk in a university research clinic. Baseline assessments were conducted over multiple days as needed to manage participant fatigue. Clinical interviews were conducted by graduate students and post-doctoral researchers with multiple years of clinical experience who were blind to participants' self-report scores. Participants were followed naturalistically for 24 months, with follow up assessments of diagnostic status conducted at 12 and 24 months. This study was observational, and participants received treatment as usual during this time from any pre-existing community providers. Participants were not enrolled in any treatment studies during this time. All procedures were approved by the university Institutional Review Board and all participants provided written informed consent.

### Measures

CHR status was assessed by the Structured Interview for Psychosis-Risk Syndromes (SIPS) (2). Psychiatric syndromes were measured by interview and self-report instruments. The broader study included a range of measures intended to capture theoretically relevant variables to psychosis, adolescent and young adult development, risk, and resilience. The authors evaluated all measures in the broader study to assess their suitability for a transdiagnostic dimensional analysis, prioritizing fit within generally accepted psychiatric syndromes based on available validity data for each scale. For instance, the Self-Rating Anxiety Scale (SAS) (34) was administered to participants, but it was not included because it primarily measures nonspecific somatic symptoms of anxiety. By contrast, the Beck Anxiety Inventory (BAI) (35) was included because multiple studies have reported that it is most closely tied to panic symptomatology (36, 37). All measures matching a given syndrome were included. When multiple measures were available for a given syndrome, all measures were included, and their mean standardized score was used as the syndrome score.

Full details including descriptive statistics for all measures are included in the Supplementary Material. Positive symptoms were assessed by the positive subscale of the SIPS, as well as the Prodromal Questionnaire-Brief (PQB) (38), the Launey-Slade Hallucination-Proneness Scale (LSHS) (39), and the positive subscale of the Community Assessment of Psychic Experiences (CAPE) (40). Negative symptoms were assessed by the negative symptom subscale of the SIPS. To achieve more equal weighting of psychotic and nonpsychotic symptoms in the factor analysis, we divided psychotic symptoms into multiple theoretically and empirically grounded symptom groups: positive-perceptual, positive-nonperceptual, negative-emotion, and negative-volition (19, 32, 41–43). See Supplementary Material 1.2 for details.

Depression was assessed by the Beck Depression Inventory (BDI-II) (44). Generalized anxiety was assessed by the generalized anxiety subscale of the Revised Screen for Child Anxiety Related Disorders (SCARED-R) (45). Social anxiety was assessed by the Social Interaction Anxiety Scale (SIAS) (46) and the social anxiety subscale of the SCARED-R. Panic was assessed by the Beck Anxiety Inventory (BAI) (35) and the panic subscale of the SCARED-R. Hypomania was assessed by the Hypomanic Personality Scale (HPS) (47) and the Responses to Positive Affect scale (RPA) (33). Substance abuse was assessed during the clinical interview as the frequency of the participant's most frequently used substance, and the impairment associated with the participant's most impairing substance. The antisocial behavior syndrome included measures of impulsivity and conduct problems. Impulsivity was assessed by the Positive Urgency Measure (PUM) (48). Conduct problems were assessed by the mean scores on the “anger,” “hate,” and “contempt” items of the Modified Differential Emotions Scale (mDES) (49); and by the occurrence of antisocial life events within the past year, assessed by a modified version of the Peri Life Events Scale (LE) (31)^2^.

Cognition was assessed by the composite score on the MATRICS Consensus Cognitive Battery (MCCB) (50). Psychosocial functioning was assessed by the Social and Role scales of the Global Functioning Scales (GFS) (51). Risk of conversion to psychosis was assessed in two ways. Cross-sectionally, we calculated baseline risk scores following the North American Prodromal Longitudinal Study procedure (52). This formula uses specific age, positive symptoms, social functioning, and cognition variables to estimate a risk for conversion within 24 months. Longitudinally, we compared baseline symptom scores in participants who converted to a confirmed psychotic disorder within 24 months vs. those who did not convert to a psychotic disorder.

### Data Analysis

Analyses were carried out in *R* version 3.6.1 (53). Baseline demographic differences between groups were compared by *chi*-squared tests (categorical data) and two-tailed independent samples *t*-tests with effect sizes expressed as Cohen's *d* (continuous data). Symptom variables were standardized to the HC participants mean and standard deviation, so that 0 indicates the HC mean and units are HC standard deviations. This allows all variables to be interpreted in the common metric of “standard deviations above/below local community norms.” Syndrome scores were calculated as the mean of standardized variables within each syndrome. We examined group differences between CHR and HC groups on syndrome scores using two-sample *t*-tests with effect sizes expressed as Cohen's *d*.

Syndrome scores were then entered into an exploratory factor analysis with minimum residual estimation and oblimin rotation, which allowed for correlated factors. The number of factors was determined by parallel analysis, which compares the eigenvalues of factors in observed data to the eigenvalues of factors in simulated random data with the same number of participants and items. To avoid overfactoring, factors were considered significant if their eigenvalues exceeded the 95th percentile of randomly simulated eigenvalues. Missing data were imputed as the median.

Factor scores were saved and compared to external clinical validators (cognition, functioning, and risk scores) by Pearson correlations. Finally, *t*-tests examined group differences in factor scores between participants who converted to a confirmed psychotic disorder vs. those who did not convert. All *p*-values were corrected for multiple comparisons using FDR-correction.

## Results

### CHR Scored Higher on All Syndromes

**Figure 1:**
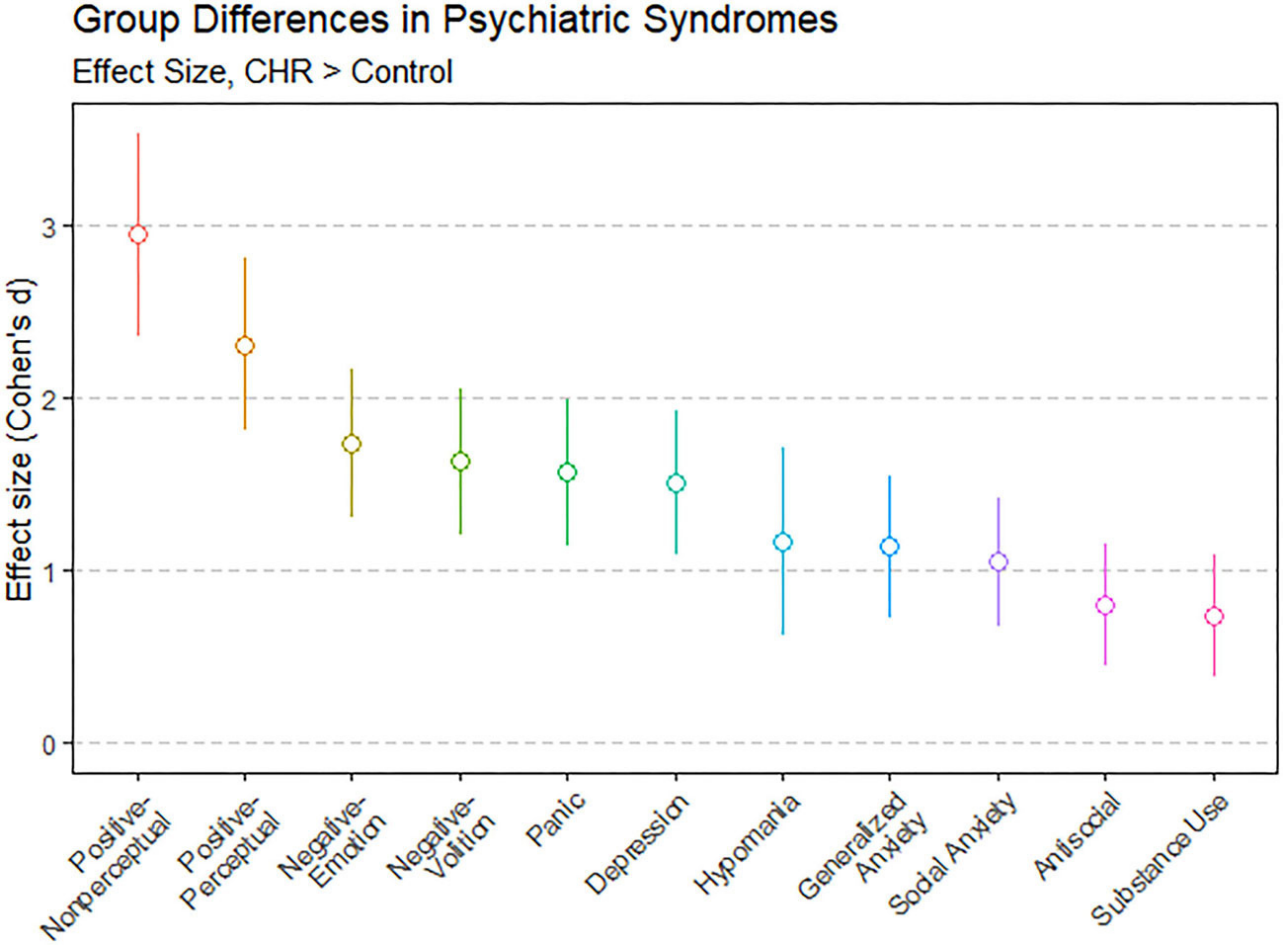
Group differences between individuals at clinical high risk for psychosis (CHR; *n* = 71) and matched control participants (*n* = 73) showed that CHR participants scored significantly higher on all psychiatric syndromes. Group differences for each syndrome are shown as effect sizes in Cohen's *d*: *d* = 0 would indicate that CHR and control means were the same, *d* = 1 would indicate that the CHR mean was 1 standard deviation above the control mean, and so on. Error bars show 95% confidence intervals. Two-tailed *t*-tests confirmed that all effects were significant, *FDR-corrected p* < 0.001.

Tests of group differences (two-tailed *t*-tests for continuous variables and chi-squared tests for categorical variables) found no significant group differences on demographic variables. As shown in Supplemental Table 1, CHR and HC groups differed on all other variables (all *p*s < 0.01) except antisocial life events (*p* = 0.060) and cognition (*p* = 0.510). For all syndromes, mean scores were higher in the CHR group than the HC group. Group differences in syndrome scores were confirmed by two-tailed *t*-tests, which found all FDR-corrected *p*-values < 0.001. Figure 1 shows effect sizes of these group differences. Effect sizes were large, ranging from *d* = 0.73 (substance use) to *d* = 2.94 (nonperceptual positive symptoms). Effect sizes were largest for positive symptoms, followed by negative symptoms, and then by other comorbid psychiatric syndromes.

### Internalizing, Negative, and Positive Psychopathology Dimensions

To examine the latent structure of syndrome scores, we carried out an exploratory factor analysis with oblique (oblimin) rotation. Parallel analysis indicated that three factors were optimal. As shown in Table 1, the three factors explained 51% of the item-level variance. Factor 1 (Internalizing) was characterized by panic, generalized anxiety, and social anxiety. Factor 2 (Negative symptoms) was characterized by avolitional and impaired emotion negative symptoms. Factor 3 (Positive symptoms) was characterized by perceptual and nonperceptual positive symptoms. Factor intercorrelations were positive and in the small to moderate range (*r*s = 0.24–0.32).

**Table 1:** Exploratory factor analysis of psychiatric syndromes in CHR group.

**Item**	**Factor 1 (Internalizing)**	**Factor 2 (Negative)**	**Factor 3 (Positive)**	**Item communality**	**Item complexity**
Panic	**0.86**	−0.12	0.07	0.68	1.1
Generalized anxiety	**0.82**	0.06	−0.06	0.69	1.0
Social anxiety	**0.53**	0.29	0.00	0.47	1.6
Negative—Volition	−0.03	**0.83**	−0.02	0.67	1.0
Negative—Emotion	−0.01	**0.78**	0.10	0.65	1.0
Depression	**0.37**	**0.48**	0.10	0.52	2.0
Positive—Non-perceptual	−0.07	0.05	**0.94**	0.88	1.0
Positive—Perceptual	0.16	0.00	**0.78**	0.69	1.1
Hypomania	−0.07	**−0.37**	**0.40**	0.24	2.1
Substance use	−0.12	−0.14	0.19	0.06	2.6
Antisocial behavior	0.22	−0.17	0.10	0.06	2.3
SS Loadings	1.98	1.89	1.74		
Proportion of variance	0.18	0.17	0.16		
Cumulative variance	0.18	0.35	0.51		
Factor intercorrelations					
Factor 2	0.32				
Factor 3	0.24	0.24			

*Bold indicates absolute factor loadings > 0.30. CHR, Clinical High Risk*.

Depression cross-loaded on the Internalizing and Negative dimensions. Hypomania cross-loaded on the Negative and Positive dimensions, with a negative loading on the Negative dimension. In other words, hypomania was associated with higher positive symptoms but lower negative symptoms. Substance use and antisocial behavior did not load onto any factors, nor did they form a separate externalizing factor. In fact, substance use and antisocial behavior were uncorrelated (*r* = −0.11, *p* = 0.37).

One possible concern with this factor analysis is its relatively low subject to item ratio (6.45:1). As a test of robustness, we dropped the substance use and antisocial behavior syndromes (due to item communalities < 0.20) and re-ran the factor analysis. This analysis, which had a somewhat higher subject to item ratio (7.89:1), found substantively identical results.

### Dimensions' Impact on Cognition, Functioning, and Conversion Risk

Did psychopathology dimensions relate to cognition and functioning? As shown in Table 2, the negative symptom dimension moderately correlated with impaired cognition, *r*_(66)_ = −0.34, *FDR-corrected p* = 0.008, and strongly correlated with impaired social, *r*_(70)_ = −0.74, *FDR-corrected p* < 0.001, and role functioning, *r*_(70)_ = −0.64, *FDR-corrected p* < 0.001. The positive symptom dimension marginally correlated with intact cognition, *r*_(66)_ = 0.21, *FDR-corrected p* = 0.079. The internalizing dimension did not correlate with cognition or functioning, all FDR-corrected *p*-values > 0.250.

**Table 2:** Psychopathology factors and clinical variables in CHR group: Pearson correlations with 95% confidence intervals.

	**Factor 1 (Internalizing)**	**Factor 2 (Negative)**	**Factor 3 (Positive)**
Cognitive function	−0.13 [−0.35, 0.12]	−0.34^**^ [−0.54, −0.11]	0.23^†^ [−0.01, 0.45]
Social function	−0.15 [−0.37, 0.09]	−0.74^***^ [−0.83, −0.61]	−0.21 [−0.42, 0.03]
Role function	−0.13 [−0.35, 0.11]	−0.64^***^ [−0.76, −0.47]	−0.09 [−0.32, 0.14]
Conversion risk score	0.22^†^ [−0.02, 0.44]	0.54^***^ [0.35, 0.69]	0.27^*^ [0.03, 0.48]

*Cognition assessed by the composite score on the MATRICS Consensus Cognitive Battery. Functioning assessed by the Global Functioning Scales. Conversion risk score calculated by the NAPLS formula*.

† *FDR-corrected p < 0.10*;

* *FDR-corrected p < 0.05*;

** *FDR-corrected p < 0.01*;

*** *FDR-corrected p < 0.001*.

Did dimensions predict risk of conversion to psychosis? We addressed this question in two ways. First, we calculated a risk score from participants' baseline data, following the NAPLS risk calculation formula (52). As shown in Table 2, the negative symptom dimension strongly correlated with risk scores, *r*_(65)_ = 0.54, *FDR-corrected p* < 0.001, the positive symptom dimension moderately correlated with risk scores, *r*_(65)_ = 0.27, *FDR-corrected p* = 0.044, and the internalizing dimension marginally correlated with risk scores, *r*_(65)_ = 0.22, *FDR-corrected p* = 0.099.

**Figure 2:**
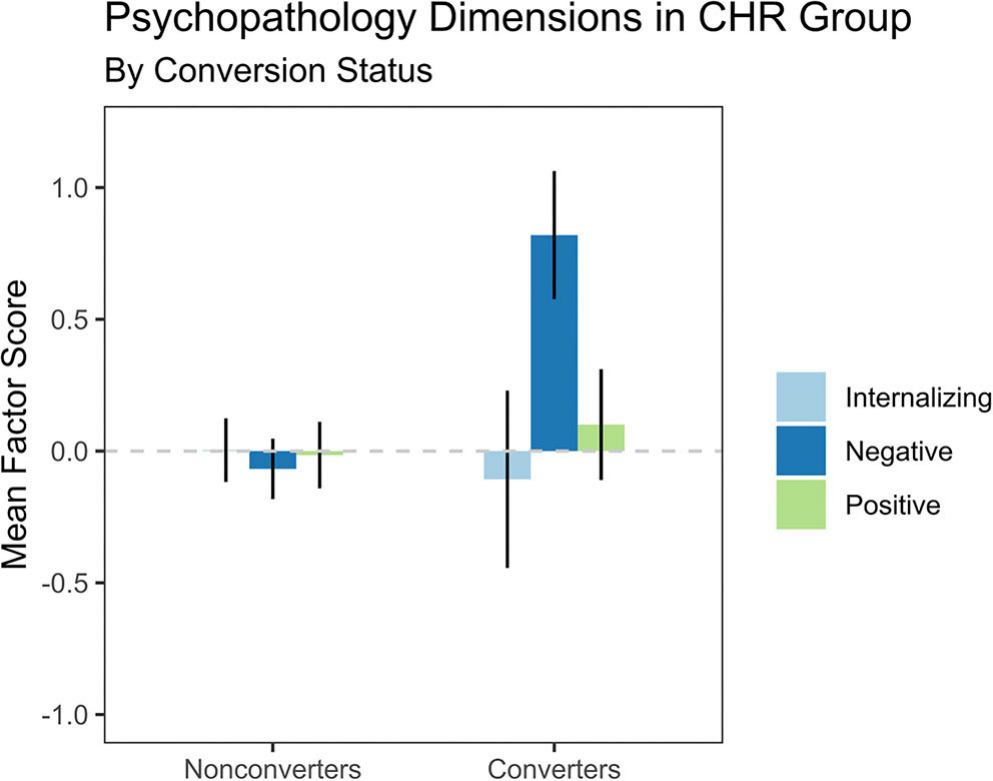
The Negative Symptoms dimension predicted conversion to psychosis in individuals at clinical high risk for psychosis (CHR). Participants who converted to a psychotic disorder within 24 months (*n* = 6) reported higher scores at baseline on Negative Symptoms, *t*_(7.43)_ = 3.30, *FDR-corrected p* = 0.036, *d* = 1.00, and no significant difference on Internalizing, *t*_(6.36)_ = −0.31, *FDR-corrected p* = 0.767, *d* = −0.12, or Positive Symptoms, *t*_(9.15)_ = 0.47, *FDR-corrected p* = 0.767, *d* = 0.12, compared to participants who did not convert to a psychotic disorder (*n* = 65). Error bars indicate the standard error of the mean.

Second, as shown in Figure 2, we compared baseline symptom dimensions in CHR participants who converted to a confirmed psychotic disorder within 24 months (*n* = 6) vs. other participants (*n* = 65). Despite the very small sample size, converters were elevated on the negative symptom dimension at baseline compared to nonconverters, with a large effect size, *t*_(7.43)_ = 3.30, *FDR-corrected p* = 0.036, *d* = 1.00. The other two dimensions did not differentiate converters from nonconverters, FDR-corrected *p*-values > 0.750.

## Discussion

This is the first study to model psychiatric comorbidity in a CHR sample in a transdiagnostic dimensional framework. The CHR state is increasingly understood as a transdiagnostic construct in clinical staging models (29, 30). Conceptually, a transdiagnostic dimensional approach is a natural fit to capture this complexity. Practically, a transdiagnostic dimensional approach can generate novel and clinically useful insights into the relationships between apparently diverse forms of psychopathology in the CHR state.

We identified eleven psychiatric syndromes in an extant dataset, including four psychotic syndromes and seven nonpsychotic syndromes. The CHR group was most elevated (compared to matched healthy controls) on positive symptoms, followed by negative symptoms, then by internalizing syndromes and hypomania, and finally by externalizing syndromes. All group differences were highly significant, and, notably, the CHR group differed from controls by more than one standard deviation on all syndromes except antisocial behavior and substance use. Individuals at CHR tended to report broad, distressing, and impactful symptoms, both psychotic and nonpsychotic.

Could latent dimensions make sense of this comorbidity picture? An exploratory factor analysis found that three dimensions accounted for a majority of the variance in syndrome scores. The first dimension captured both fearful (panic, social anxiety) and distressed (generalized anxiety, depression) internalizing. The second dimension captured primarily negative symptoms, and the third dimension captured primarily positive symptoms. As predicted by hierarchical models such as HiTOP, the three dimensions corresponded to three higher-order psychopathology spectra (internalizing, detachment, and thought disorder) and positively correlated with one another with small to medium effect sizes (*r* = 0.24–0.32).

The negative symptom factor was much more strongly linked than the other factors to impaired cognition, impaired social and role functioning, and risk of conversion to psychosis. In fact, the only significant correlation for another factor was between positive symptoms and the conversion risk score. This correlation is slightly dubious because the NAPLS risk score includes two positive SIPS items in its risk calculation formula, which makes the positive symptom and risk score variables slightly statistically dependent. Moreover, despite the very small sample size of converters, the negative symptoms factor prospectively predicted conversion while the positive symptoms factor did not. Multiple studies have shown that negative symptoms are predictive of conversion to psychosis (54–56). The current study strengthens those findings. The approach in this study—exploratory modeling of multivariate dimensions—is novel, and it is noteworthy that this analysis confirmed the importance of negative symptoms. This effect seems to be robust to very different statistical methodologies.

Moreover, several nuances of the factor structure provide novel insights into comorbidity in the CHR state. Help-seeking individuals meeting CHR criteria have been described as “a troubled group presenting with many comorbid problems” (5), and it is critical to understand how these problems interact to predict which individuals will go on to develop psychotic disorders and which will go on to develop chronic nonpsychotic disorders. The current study found several novel insights into CHR comorbidity: depression and hypomania were hybrid constructs, and externalizing syndromes (substance use and antisocial behavior) failed to load on any factors.

Depression was a hybrid construct, loading onto both internalizing and negative symptoms. The detachment and internalizing spectra are generally found to be distinct in adult clinical populations (15); however, multiple studies have found considerable overlap between depression and negative symptoms in CHR samples (57–59). Incipient negative symptoms can closely resemble internalizing symptoms, and depressed mood is often one of the earliest observable signs of a high risk syndrome (29, 60). The current study adds to this body of research by showing that, even at the level of broad dimensions, self-reported depression can indicate internalizing, detachment, or both. Notably, this finding was specific to depression; self-reported measures of generalized anxiety, social anxiety, and panic were clearly separate from negative symptoms. One practical implication concerns research which statistically controls for depression when studying negative symptoms [e.g., (61)]. If depression and negative symptoms partly form a common psychopathology factor, then statistically controlling for depression will reduce the effects of negative symptoms in unpredictable ways. Further research is required to determine the dividing lines, if any, between depression and negative symptoms in the CHR state.

Hypomania was another hybrid construct, as a component of higher positive symptoms but lower negative symptoms. There is some debate about transdiagnostic relationships between mania, thought disorder, and internalizing: is mania a component of internalizing, a component of thought disorder, a blend of both, or a separate dimension entirely (15, 20, 22, 23, 62)? This study suggests a somewhat novel placement of (hypo)mania in the CHR state, as a component of thought disorder that in some way protects against detachment. Perhaps individuals who tend toward mania and grandiosity would experience lower-intensity psychotic-like experiences as being highly salient, meaningful, and personally significant—for example, as evidence that the individual has been chosen for a special purpose by a higher power. These individuals might report significant positive symptoms, without attendant negative symptoms. Crucially, because the negative symptom dimension was most associated with risk of psychotic disorders, a hypomanic-positive symptom presentation with low negative symptoms would be less likely to indicate an incipient psychotic disorder. This intriguing possibility warrants follow-up research examining (hypo)mania's prognostic role in the CHR state.

Externalizing syndromes—substance use and antisocial behavior—did not load onto any of the three factors. Nor did they form an externalizing factor—in fact, they were uncorrelated (*r* = −0.11, *p* =0.37). This negative result is difficult to interpret, given that the quality of the variables was generally poorest for these syndromes. The CHR state has not traditionally been associated with externalizing and, like most CHR studies, the broader study from which these data were drawn did not focus on externalizing. It may be worth attending more to externalizing in CHR studies. A recent review has shown that childhood antisocial and aggressive behavior predicts later psychotic symptoms, suggesting that there may be an unrecognized link between externalizing and the CHR state (63). Future research taking a transdiagnostic dimensional approach in the CHR state would be enhanced by more complete assessment of externalizing syndromes.

We consider this study to be preliminary because of two notable limitations. First, the study was limited by sample size. The subject to item ratio was less than ideal for factor analysis. A supplemental analysis improved the subject to item ratio by dropping the substance use and antisocial behavior syndromes and found similar results; nevertheless, the sample size could have caused misclassifications in the factor solution. The sample size of converters was also very small (*n* = 6) in the prospective analysis of conversion risk (Figure 2), and these results are speculative. It would be valuable to examine the transdiagnostic structure of psychopathology in larger CHR samples, particularly as CHR samples may contain individuals in multiple clinical stages of disorder pathogenesis, in which symptoms may exhibit different latent structures (29). Future research would be particularly valuable to comparing transdiagnostic structure between clinical stages. Second, the study was limited in its coverage of externalizing and personality. The HiTOP model posits that psychopathology spectra correspond to normative and pathological personality dimensions (15, 20, 25), but we were unable to validate factors with respect to personality because the dataset contained no personality measures. Future research on transdiagnostic dimensional models of psychopathology in the CHR state could build on these preliminary findings by including larger sample sizes and more comprehensive assessment including measures of externalizing and personality traits. Other potential limitations include possible effects of participant fatigue and treatment by community healthcare providers. Ultimately, no one factor solution is ever definitive, and our goal in presenting this study is to stimulate further research with other datasets—which will have their own strengths and weaknesses—to continue defining the dimensional contours of comorbidity in CHR populations.

## Data Availability Statement

The data analyzed in this study is subject to the following licenses/restrictions: Dataset not publicly available due to sensitivity of the clinical data and risks of deidentification. Requests to access these datasets should be directed to Henry R. Cowan, hrcowan@u.northwestern.edu.

## Ethics Statement

The studies involving human participants were reviewed and approved by University of Colorado-Boulder Institutional Review Board. The patients/participants provided their written informed consent to participate in this study.

## Author Contributions

HC contributed to study design, data analysis, and manuscript writing. VM contributed to study design, data collection, and manuscript writing. All authors contributed to the article and approved the submitted version.

## Conflict of Interest

The authors declare that the research was conducted in the absence of any commercial or financial relationships that could be construed as a potential conflict of interest.

## Footnotes

^1^“Syndrome” and “disorder” have slightly different meanings in this literature. “Syndrome” refers to an empirical group of symptoms/traits which cluster together. Traditionally, “disorder” refers not only to a psychiatric syndrome but also to specific diagnostic criteria, prevalence, course, subtypes, specifiers, and putative mechanisms (11, 15). Because these concepts are closely related, the study of syndromes can greatly enhance our understanding of traditional disorders. In this paper, we refer to our main variables as syndromes because they are defined psychometrically rather than diagnostically (15).

^2^See Supplemental Material 1.1 for details on antisocial life events.
