# Supplementary material for: Transdiagnostic Dimensions of Psychiatric Comorbidity in Individuals at Clinical High Risk for Psychosis: A Preliminary Study Informed by HiTOP
Source: Front Psychiatry. 2021 Jan 8;11:614710. doi: 10.3389/fpsyt.2020.614710 (PMC7819881; doi:10.3389/fpsyt.2020.614710)
Supplement: Supplementary file 1 [file Data_Sheet_1.DOCX]

Supplementary Material

# Supplementary Data

## Full Descriptions of Study Measures

**Structured Interview for Psychosis-Risk Syndromes (SIPS):** The SIPS is a semistructured clinical interview that assesses positive (e.g., perceptual abnormalities), negative (e.g., emotional expression), disorganized (e.g., personal hygiene), and general symptoms (e.g., sleep disturbance), with a total score calculated for each category. It is one of the most widely used assessments of the clinical high risk for psychosis syndrome. This study included the positive and negative subscales of the SIPS.

**Prodromal Questionnaire-Brief (PQB):** The PQB is a 21-item measure of unusual thought content, suspiciousness, grandiosity, perceptual abnormalities, and disorganized communication.

**Launay-Slade Hallucination-Proneness Scale (LSHS):** The LSHS is a 12-item, unidimensional measure of predisposition toward perceptual hallucinations.

**Community Assessment of Psychic Experiences (CAPE):** The CAPE is a 42-item self-report measure of psychotic-like experiences (PLEs), divided into positive, negative, and depressive dimensions [16]. The positive subscale contains 20 items, each of which is rated for frequency (1 = “Never” to 4 = “Nearly always”) and distress (1 = “Not distressed” to 4 = “Very distressed”). The frequency items on the CAPE positive subscale were used in this study, as they are more commonly used in CHR research.

**Beck Depression Inventory (BDI-II):** The BDI is a commonly used self-report scale for depression. It consists of 21 items assessing DSM-IV depressive symptoms (e.g., sadness and irritability). Each item is rated from not present (0) to severe (3), and a total score indicates the severity of depressive symptomatology.

**Revised Screen for Child Anxiety Related Disorders (SCARED-R):** The SCARED-R is a 41-item questionnaire developed to assess the presence of anxiety in children 8 and older. Although the SCARED-R has subscales assessing eight forms of anxiety, we chose not to include all subscales, as this would have produced many syndromes in the fearful internalizing spectrum based on subscales of this one instrument. Therefore, the current study only included the SCARED-R subscales for social anxiety, panic, and generalized anxiety.

**Social Interaction Anxiety Scale (SIAS):** The SIAS is a 19-item questionnaire assessing fear of social interactions.

**Beck Anxiety Inventory (BAI):** The BAI is a 21-item self-report measure of anxiety symptom severity, with each item rated from not present (0) to severe (3), and a total score indicating the severity of anxious symptomatology. Although the BAI was developed as a nonspecific measure of anxiety, multiple studies have shown that it primarily relates to symptoms of panic.

**Hypomanic Personality Scale (HPS):** The HPS is a 48-item self-report measure of tendencies toward hypomania and risk for future manic episodes.

**Responses to Positive Affect scale (RPA):** The RPA is a 17 item self-report measure of rumination and dampening in response to positive affect, which commonly occur in hypomania and mania.

**Positive Urgency Measure (PUM):** The PUM is a 14-item self-report measure of tendencies to engage in risky, impulsive, or harmful behavior when feeling positive emotion.

**Modified Differential Emotions Scale (mDES):** The mDES is an 18-item measure of positive and negative affect, which asks participants to report how frequently they experience various emotions. For this study, we examined only the items for “anger”, “hate”, and “contempt” as indices of dysregulated externalizing emotional experiences.

**Peri Life Events Scale (LE):** The LE inventory is a 59-item inventory of life events, which asks participants to endorse whether they have experienced each event in the last year. For this study, we examined the mean occurrence antisocial life events in the past year, defined as: bullied or got in a fight; involved in an accident, lawsuit, or court case; accused of a crime; lost driver’s license; arrested; went to jail; convicted or acquitted of a crime; released from jail; didn’t get out of jail when expected.

**MATRICS Consensus Cognitive Battery (MCCB):** The MCCB is a standardized battery of cognitive assessments designed for schizophrenia populations, consisting of 10 tests assessing seven cognitive domains (processing speed, attention/vigilance, working memory, verbal learning, visual learning, problem solving, and social cognition).

**Global Functioning Scales (GFS):** The GFS are interview-rated assessments of social and role functioning. Based on a standardized set of assessment questions, the clinical interviewer assigns ratings from 0 (lowest) to 10 (highest) to indicate the participant’s current level of real-world social and role functioning.

## Psychotic symptom groupings

To achieve more equal weighting of psychotic and nonpsychotic symptoms in the factor analysis, we divided psychotic symptoms into multiple theoretically grounded symptom groups.

For positive symptoms, we calculated scores for perceptual positive symptoms (e.g., hallucinations) and nonperceptual positive symptoms (e.g., delusions, paranoia). This distinction aligns with factor analyses of the PQB (1) and CAPE (2) as well as the CAARMS, another widely-used interview measure of the CHR state (3). The positive-perceptual score included the SIPS perceptual abnormalities item (P4), the LSHS, and the perceptual abnormalities factors from the PQB and CAPE (1,2). The positive-nonperceptual score included the SIPS unusual thought content (P1), suspiciousness (P2), and grandiosity (P3) items, the PQB “grandiose/unusual delusions” and “persecutory/thought delusions” factors, and the CAPE “bizarre experiences” and “persecutory ideation” factors.

For negative symptoms, we calculated scores for avolitional negative symptoms and loss of emotion negative symptoms. This distinction is supported by a factor analytic study of SIPS negative symptom data (4), and conforms to a broad distinction in psychosis research between apathy/asociality and inexpressivity/flat affect (5). The negative-volition score included SIPS avolition (N2) and occupational functioning (N6), while the negative-emotion score included SIPS expression of emotion (N3), experience of emotion and self (N4), and social anhedonia (N1).

# Supplementary Figures and Tables

| *Table S1.*  Unstandardized Descriptive Statistics and Group Comparisons for all Study Variables | | | | | | | | | | | | | | | |  |  |
| --- | --- | --- | --- | --- | --- | --- | --- | --- | --- | --- | --- | --- | --- | --- | --- | --- | --- |
|  |  | | |  | | CHR | | | |  | | HC | | |  | |  |
| Syndrome | | Variable | Mean | | SD | |  | Mean | | | SD | | *t* | *p* | | | |
| Positive symptoms | | SIPS-Positive | 2.40 | | 0.91 | |  | 0.09 | 0.21 | | | | 21.0 | <.001 | | | |
|  | | PQB | 1.56 | | 0.94 | |  | 0.19 | 0.31 | | | | 11.6 | <.001 | | | |
|  | | LSHS | 1.73 | | 0.83 | |  | 0.52 | 0.54 | | | | 10.3 | <.001 | | | |
|  | | CAPE-Positive | 0.67 | | 0.37 | |  | 0.22 | 0.18 | | | | 8.98 | <.001 | | | |
| Negative symptoms | | SIPS-Negative | 1.65 | | 1.17 | |  | 0.07 | 0.16 | | | | 11.4 | <.001 | | | |
| Depression | | BDI | 0.82 | | 0.55 | |  | 0.19 | 0.24 | | | | 8.76 | <.001 | | | |
| Generalized anxiety | | SCARED-GAD | 1.04 | | 0.46 | |  | 0.52 | 0.46 | | | | 6.21 | <.001 | | | |
|  | |  |  | |  | |  |  |  | | | |  |  | | | |
| Social anxiety | | SIAS | 1.42 | | 0.87 | |  | 0.61 | 0.49 | | | | 6.68 | <.001 | | | |
|  | | SCARED-Soc | 0.87 | | 0.61 | |  | 0.45 | 0.48 | | | | 4.23 | <.001 | | | |
| Panic | | BAI | 0.88 | | 0.54 | |  | 0.25 | 0.30 | | | | 9.18 | <.001 | | | |
|  | | SCARED-Panic | 0.51 | | 0.40 | |  | 0.11 | 0.18 | | | | 7.19 | <.001 | | | |
|  | |  |  | |  | |  |  |  | | | |  |  | | | |
| Hypomania | | HPS | 0.49 | | 0.17 | |  | 0.30 | 0.15 | | | | 5.05 | <.001 | | | |
|  | | RPA | 2.34 | | 0.42 | |  | 2.05 | 0.42 | | | | 2.83 | .006 | | | |
| Substance use | | Frequency | 2.33 | | 1.75 | |  | 1.22 | 1.48 | | | | 4.08 | <.001 | | | |
|  | | Impairment | 2.02 | | 0.83 | |  | 1.54 | 0.56 | | | | 3.94 | <.001 | | | |
| Antisocial behavior | | PUM | 2.22 | | 0.47 | |  | 1.47 | 0.43 | | | | 6.89 | <.001 | | | |
|  | | mDES^a^ | 2.20 | | 0.60 | |  | 1.73 | 0.59 | | | | 3.33 | .001 | | | |
|  | | Antisocial life events | 0.08 | | 0.12 | |  | 0.04 | 0.11 | | | | 1.89 | .060 | | | |
| Cognition | | MCCB composite | 45.94 | | 9.91 | |  | 44.87 | 9.05 | | | | 0.66 | .510 | | | |
| Functioning | | GFS-Social | 6.62 | | 1.72 | |  | 8.72 | 0.63 | | | | -9.74 | <.001 | | | |
|  | | GFS-Role | 6.81 | | 1.69 | |  | 8.57 | 0.67 | | | | -8.22 | <.001 | | | |
| *Note:* ^a^Anger, hate, and contempt items.  HiTOP = Hierarchical taxonomy of psychopathology; CHR = Clinical high risk for psychosis; HC = Healthy Comparison; SIPS = Structured Interview for Psychosis-Risk Syndromes; PQB = Prodromal Quesitonnaire-Brief; LSHS = Launey-Slade Hallucination-Proneness Scale; CAPE = Community Assessment of Psychic Experiences; BDI = Beck Depression Inventory-II; SCARED = Screen for Child Anxiety Related Disorders; SIAS = Social Interaction Anxiety Scale; BAI = Beck Anxiety Inventory; HPS = Hypomanic personality scale; RPA = Responses to Positive Affect Scale; PUM = Positive Urgency Measure; mDES = Modified Differential Emotions Scale; MCCB = MATRICS Consensus Cognitive Battery; GFS = Global Functioning Scales. | | | | | | | | | | | | | | | |  |  |

**References**

1. Azis M, Rouhakhtar PR, Schiffman JE, Ellman LM, Strauss GP, Mittal VA. Structure of positive psychotic symptoms in individuals at clinical high risk for psychosis. *Early Intervention in Psychiatry* (2020)1–8.

2. Cowan HR, Mittal VA. Three types of psychotic-like experiences in youth at clinical high risk for psychosis. *Eur Arch Psychiatry Clin Neurosci* (2020) doi:10.1007/s00406-020-01143-w

3. Yung AR, Yuen HP, McGorry PD, Phillips LJ, Kelly D, Dell’Olio M, Francey SM, Cosgrave EM, Killackey E, Stanford C, et al. Mapping the onset of psychosis: the Comprehensive Assessment of At‐Risk Mental States.8.

4. Azis M, Strauss GP, Walker E, Revelle W, Zinbarg R, Mittal V. Factor Analysis of Negative Symptom Items in the Structured Interview for Prodromal Syndromes. *Schizophr Bull* (2018) doi:10.1093/schbul/sby177

5. Kotov R, Foti D, Li K, Bromet EJ, Hajcak G, Ruggero CJ. Validating dimensions of psychosis symptomatology: Neural correlates and 20-year outcomes. *Journal of Abnormal Psychology* (2016) **125**:1103–1119. doi:10.1037/abn0000188
